# Dataset of methane pyrolysis products in a batch reactor as a function of time at high temperatures and pressures

**DOI:** 10.1016/j.dib.2023.108953

**Published:** 2023-02-09

**Authors:** James Tatum, Ambuj Punia, Larry Kostiuk, Marc Secanell, Jason Olfert

**Affiliations:** a University of Alberta, 116 St & 85 Ave, Edmonton, AB T6G 2R3 Canada; b Carleton University, 1125 Colonel By Dr, Ottawa, ON K1S 5B6 Canada

**Keywords:** Methane decomposition, Hydrogen production, Constant-volume reactor, Green technology, CO_2_ free

## Abstract

Methane pyrolysis is a process used to generate hydrogen gas and carbon black without the creation of carbon dioxide. Methane pyrolysis in a constant volume batch reactor was investigated at temperatures of 892, 1093, and 1292 K with reaction times of 15, 30, 60, 180, and 300 s at an initial pressure of 399 kPa. A quartz vessel (32 mL) was placed inside an oven where it was heated to high temperatures. At the beginning of the process, the quartz vessel was vacuumed, then flushed with nitrogen before being vacuumed again prior to every experiment. Pressurized methane was then injected into the vessel for an allocated reaction time and collected in a sample bag post reaction for analysis. The molar concentration of the product gas was analyzed using gas chromatography. Hydrogen molar concentration increased as temperature and reaction time increased. For experiments completed at 892 K the hydrogen molar concentration varied from 10.0 ± 5.9% with a 15 s reaction time to 26.5 ± 0.8% for a 300 s reaction time. For experiments completed at 1093 K the hydrogen molar concentration varied from 21.8 ± 3.7% for a 15 s reaction time to 53.0 ± 2.9% for a 300 s reaction time. For experiments completed at 1292 K the hydrogen molar concentration varied from 31.5 ± 1.7% for a 15 s reaction time to 53.0 ± 2.4% for a 300 s reaction time.

**Specifications Table**

**Table:** 

Subject	Energy
Specific subject area	Methane pyrolysis via thermal decomposition
Type of data	Table
How the data were acquired	Gas Chromatography in combination with sample bags were used to determine the molar concentration of the product species. The sample bag was vacuumed before being filled with the products after an experiment was completed. This sample bag was then connected to the Gas Chromatograph (GC) and the product gas was run through the GC method that shows the molar concentration of the product gas.
Data format	Analyzed, Raw
Description of data collection	Experiments were run with a quartz vessel in a furnace set at 873, 1073, and 1273 K with the regulator on the methane bottle (99.9% methane and 0.1% helium) set at 400 kPa. The actual measured temperatures inside the vessel were 892 ± 5 K, 1093 ± 6 K, and 1292 ± 8 K with an initial pressure of 399 ± 4 kPa. (The uncertainties represent 95% confidence intervals of the total uncertainty including bias and precision uncertainty). Several different reaction times were analyzed for each temperature to determine the gas-phase products at each. At each temperature the reaction times of 15, 30, 60, 180, and 300 s were investigated. Solid carbon deposition on the quartz tube was not measured for these experiments due to the difficulty of getting an accurate measurement. All carbon was removed in between experiments. For a sample to be considered acceptable they had to have a total molar concentration sum of 95% or greater with no more than 5% of non-reactive species (Nitrogen, Oxygen, Carbon Dioxide, and Carbon Monoxide). The non-reactive species were removed from the total molar concentration to normalize the data (see attached excel file for formula). This was done because the quartz vessel had such a small volume (32 mL), and it was almost impossible to remove all the other contents from the sample bag with the vacuum pump before each experiment.
Data source location	• Institution: University of Alberta • City/Town/Region: Edmonton, Alberta • Country: Canada
Data accessibility	Repository name: Mendeley Data Data identification number: doi:10.17632/3d4yftz66n.1[1] Direct URL to data: https://data.mendeley.com/datasets/3d4yftz66n

**Value of the Data**

• This data expands the methane pyrolysis experimental results to include the decomposition products at high pressures and low temperatures that were not previously investigated, especially for large residence times.
• Kinetic models for methane pyrolysis can be improved by including this dataset and the models can work for wider reactor conditions including higher pressure reactions that were not previously investigated.
• Engineers and scientists who want to learn more about the effects of high pressure and low temperature on methane decomposition chemistry.
• This data can be recreated and validated in other experiments and slight changes can be made to better understand the effect pressure, temperature, and reaction time have for further methane pyrolysis research.

## 1 Objective

This data was generated because there is no experimental data for methane pyrolysis experiments at higher pressures in literature (all literature data is around atmospheric pressure). This data expands the data set available for creating numerical models and gives future experimentalists more data to compare their results to. This data was also generated because there is no experimental data at low temperatures (less than 950 K) in literature. This further expands available experimental data to assist modelers and experimentalists.

## 2 Data Description

The dataset consists of Table 1 – Table 10 in the referenced Mendeley Data titled: “Methane pyrolysis products in a constant-volume reactor as a function of time”. All these tables show the experimental results described in detail below.

Table 1 presents the results of the experiments completed with the furnace set at 873 K with an initial pressure of 399 kPa and this is all the raw data collected from the Gas Chromatograph (GC) as well as the scaling factor, average temperature measured throughout the experiment, and the final pressure just before it was exhausted into the sample bag.

Table 2 presents the normalized data from Table 1.

Table 3 presents the results of the experiments completed with the furnace set at 1073 K with an initial pressure of 399 kPa and this is all the raw data collected from the GC as well as the scaling factor, average temperature measured throughout the experiment, and the final pressure just before it was exhausted into the sample bag.

Table 4 presents the normalized data from Table 3.

Table 5 presents the results of the experiments completed with the furnace set at 1273 K with an initial pressure of 399 kPa and this is all the raw data collected from the GC as well as the scaling factor, average temperature measured throughout the experiment, and the final pressure just before it was exhausted into the sample bag.

Table 6 presents the normalized data from Table 5.

Table 7 presents the average molar concentration for the products of experiments completed for reaction times of 15, 30, 60, 180, and 300 s at a temperature of 892 K and an initial pressure of 399 kPa.

Table 8 presents the average molar concentration for the products of experiments completed for reaction times of 15, 30, 60, 180, and 300 s at a temperature of 1093 K and an initial pressure of 399 kPa.

Table 9 presents the average molar concentration for the products of experiments completed for reaction times of 15, 30, 60, 180, and 300 s at a temperature of 1292 K and an initial pressure of 399 kPa.

Table 10 shows the average final pressures and their uncertainties for the experiments completed at 892, 1093, and 1292 K for reaction times 15, 30, 60, 180, and 300 s.

**Fig. 1:**
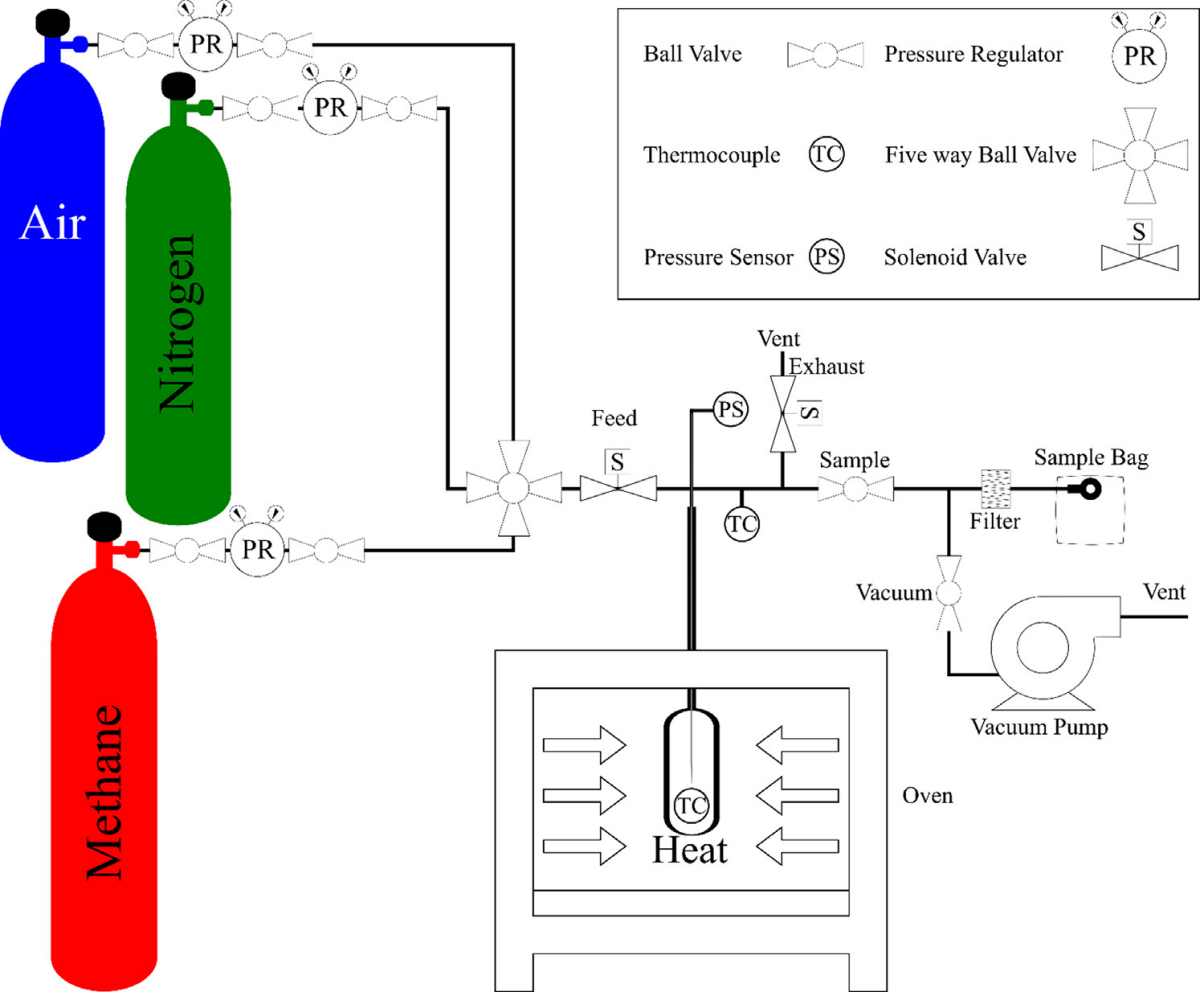
Batch reactor experimental setup used for methane pyrolysis.

The dataset also consists of Fig. 1 and Tables A1–A5 in this Data in Brief article titled: “Dataset of methane pyrolysis products in a batch reactor as a function of time at high temperatures and pressures.” These tables are for additional information and do not contain experimental results but contain additional details in how the results were determined. Such as information on error calculation and GC information such as calibration.

Fig. 1. shows the experimental setup.

Table A1. shows the calculated limit of detection for hydrocarbons measured by the Gas Chromatograph.

**Table A1:** Limit of detection for low-concentration hydrocarbons.

Species	$L_{D}$ (mole%)	$\sigma_{L}$ (mole%)	Number of Calibrations	Calibration Gas Concentration (mole%)
Ethane	1.83 × 10^−5^	1.11 × 10^−5^	4	0.001
Propane	2.61 × 10^−5^	1.59 × 10^−5^	4	0.001
Ethylene	4.20 × 10^−5^	2.55 × 10^−5^	3	0.00508
Acetylene	7.40 × 10^−5^	4.50 × 10^−5^	3	0.00512
Benzene	8.94 × 10^−5^	5.44 × 10^−5^	3	0.00512

Table A2. shows the maximum relative difference for every species the Gas Chromatograph is calibrated for.

**Table A2:** Maximum relative difference for the GC for every measured species.

Species	E
Helium	0.021
Hydrogen	0.0033
Methane	0.028
Ethane	0.060
Ethylene	0.059
Propane	0.046
CO_2_	0.053
O_2_	0.056
Nitrogen	0.069
Acetylene	0.105
CO	0.029
Benzene	0.060

Table A3. shows the columns used in Gas Chromatograph.

**Table A3:** Columns used in GC (as seen in Falahati [4])

Column number	Length (m)	Diameter (mm)	Model Number
1	60	0.25	CP8780
2	0.5	2	G3591-81023
3	1.83	2	G3591-81037
4	2.44	2	G3591-80022
5	0.91	2	G3591-81135
6	1.83	2	G3591-81035

Table A4. shows the calibration information of the Gas Chromatograph.

**Table A4:** GC calibration data.

Compound	Time window (min)	Retention Time (min)	Standard #	Molar Concentration (%)	Area	Response Factor
Helium	0.610 - 0.709	0.657	13	0.02	30.98	6.45575 × 10^−4^
			15	0.05	75.15	6.65362 × 10^−4^
			19	0.10	151.79	6.58810 × 10^−4^
			14	0.1006	152.81	6.58330 × 10^−4^

Hydrogen	0.710 - 1.050	0.788	5	4.005	10129.00	3.95443 × 10^−4^
			6	19.93	50538.20	3.94355 × 10^−4^
			18	50.44	127412.00	3.95880 × 10^−4^

Methane	1.106 - 1.264	1.144	15	0.001	2.29	4.36698 × 10^−4^
			5	0.9947	2209.10	4.50275 × 10^−4^
			6	4.971	11658.80	4.26373 × 10^−4^
			18	49.56	114784.00	4.31768 × 10^−4^
			13	49.97	115522.00	4.32559 × 10^−4^
			12	94.96	216317.00	4.38985 × 10^−4^
			19	99.9	226873.00	4.40334 × 10^−4^

Ethane	1.308-1.602	1.42	15	0.001	4.29	2.33053 × 10^−4^
			5	1.013	4251.83	2.38251 × 10^−4^
			12	2.4	10843.90	2.21323 × 10^−4^
			6	4.994	22304.40	2.23902 × 10^−4^

Ethylene	1.830 - 1.985	1.937	17	0.00508	22.82	2.22572 × 10^−4^
			16	0.102	432.80	2.35677 × 10^−4^

Propane	2.697 - 3.147	2.959	15	0.001	6.54	1.52889 × 10^−4^
			12	0.0589	404.78	1.45511 × 10^−4^
			5	1.008	6387.74	1.57802 × 10^−4^
			6	5.07	33730.10	1.50311 × 10^−4^

CO_2_	3.270 - 4.070	3.67	12	0.8108	1431.28	5.66486 × 10^−4^
			1	5.01	9008.28	5.56155 × 10^−4^
			10	10.01	18321.90	5.46340 × 10^−4^
			9	19.95	37438.60	5.32873 × 10^−4^

O_2_	5.548 - 5.833	5.653	3	0.1005	178.96	5.61585 × 10^−4^
			1	1.01	1617.02	6.24607 × 10^−4^
			10	3.992	6746.77	5.91691 × 10^−4^
			9	19.99	33911.50	5.89476 × 10^−4^

Nitrogen	5.904 - 6.488	6.065	12	1.76	3506.68	5.01900 × 10^−4^
			13	49.9	95689.80	5.21477 × 10^−4^
			6	65.035	121861.00	5.33683 × 10^−4^
			10	85.396	158727.00	5.38005 × 10^−4^
			5	92.979	172454.00	5.39151 × 10^−4^
			15	99.906	186462.00	5.35797 × 10^−4^
			17	99.98468	185418.00	5.39239 × 10^−4^

Acetylene	6.820 - 8.392	7.336	17	0.00512	24.88	2.05748 × 10^−4^
			16	0.102	554.22	1.84043 × 10^−4^

CO	7.610 - 9.110	8.11	1	0.0998	196.21	5.08651 × 10^−4^
			10	0.6016	1178.73	5.10380 × 10^−4^
			9	3.005	5964.02	5.03855 × 10^−4^
			11	9.983	19404.60	5.14467 × 10^−4^
			2	19.95	38356.80	5.20117 × 10^−4^

Benzene	16.905-18.106	17.406	17	0.00512	68.28	7.49817 × 10^−5^
			16	0.101	1270.77	7.94797 × 10^−5^

Table A5. shows the relative differences for all measured species of the Gas Chromatograph (E is the maximum relative difference).

**Table A5:** GC relative differences for all measured species (E is the maximum relative difference).

Compound	Standard #	Number of tests	Standard Molar Concentration (%)	Area	Equation from calibration curve	Measured Concentration (%)	Relative Difference	E
Helium	13	5	0.02	30.98	1517.29	0.02042	0.020902	0.0209
	15	4	0.05	75.15	1517.29	0.04953	0.009459	
	19	6	0.10	151.79	1517.29	0.10004	0.000393	
	14	3	0.1006	152.81	1517.29	0.10071	0.001122	

Hydrogen	5	5	4.005	10129.00	2527.35	4.00776	0.000689	0.0033
	6	5	19.93	50538.20	2527.35	19.99655	0.003339	
	18	5	50.44	127412.00	2527.35	50.41335	0.000528	

Methane	15	4	0.001	2.29	2282.57	0.00100	0.003215	0.0275
	5	5	0.9947	2209.10	2282.57	0.96781	0.027033	
	6	5	4.971	11658.80	2282.57	5.10775	0.027509	
	18	5	49.56	114784.00	2282.57	50.28715	0.014672	
	13	5	49.97	115522.00	2282.57	50.61047	0.012817	
	12	5	94.96	216317.00	2282.57	94.76900	0.002011	
	19	6	99.9	226873.00	2282.57	99.39361	0.005069	

Ethane	15	4	0.001	4.29	4466.98	0.00096	0.039423	0.0604
	5	5	1.013	4251.83	4466.98	0.95183	0.060381	
	12	5	2.4	10843.90	4466.98	2.42757	0.011486	
	6	5	4.994	22304.40	4466.98	4.99317	0.000166	

Ethylene	17	3	0.00508	22.82	4243.71	0.00538	0.058725	0.0587
	16	3	0.102	432.80	4243.71	0.10199	0.000146	

Propane	15	4	0.001	6.54	6640.90	0.00098	0.015091	0.0458
	12	5	0.0589	404.78	6640.90	0.06095	0.034852	
	5	5	1.008	6387.74	6640.90	0.96188	0.045755	
	6	5	5.07	33730.10	6640.90	5.07915	0.001804	
CO_2_	12	5	0.8108	1431.28	1863.87	0.76791	0.052902	0.0529
	1	4	5.01	9008.28	1863.87	4.83310	0.035309	
	10	4	10.01	18321.90	1863.87	9.83002	0.017980	
	9	4	19.95	37438.60	1863.87	20.08647	0.006841	

O_2_	3	4	0.1005	178.96	1695.95	0.10552	0.049958	0.0560
	1	4	1.01	1617.02	1695.95	0.95346	0.055981	
	10	4	3.992	6746.77	1695.95	3.97817	0.003465	
	9	4	19.99	33911.50	1695.95	19.99560	0.000280	

Nitrogen	12	5	1.76	3506.68	1863.66	1.88161	0.069097	0.0691
	13	5	49.9	95689.80	1863.66	51.34519	0.028962	
	6	5	65.035	121861.00	1863.66	65.38812	0.005430	
	10	4	85.396	158727.00	1863.66	85.16966	0.002651	
	5	5	92.979	172454.00	1863.66	92.53528	0.004772	
	15	4	99.906	186462.00	1863.66	100.05169	0.001458	
	17	3	99.98468	185418.00	1863.66	99.49150	0.004933	

Acetylene	17	3	0.00512	24.88	5432.08	0.00458	0.105258	0.1053
	16	3	0.102	554.22	5432.08	0.10203	0.000265	

CO	1	4	0.0998	196.21	1927.93	0.10177	0.019740	0.0294
	10	4	0.6016	1178.73	1927.93	0.61140	0.016286	
	9	4	3.005	5964.02	1927.93	3.09349	0.029448	
	11	4	9.983	19404.60	1927.93	10.06501	0.008215	
	2	5	19.95	38356.80	1927.93	19.89536	0.002739	

Benzene	17	3	0.00512	68.28	12583.77	0.00543	0.059825	0.0598
	16	3	0.101	1270.77	12583.77	0.10098	0.000154	

## 3 Experimental Design, Materials and Methods

A diagram of the batch reactor used in the experimental setup is shown in Fig. 1. A quartz vessel (internal volume of 32 mL) was placed inside a muffle furnace (Thermo Scientific Type 47900) where it was heated to high temperatures. The temperature and pressure of the gas inside the vessel were measured with a thermocouple (Omega TJ36-CAIN-116E-18) and absolute pressure sensor (SSI Technologies Inc. P51-100-G-B-I36-5V-000-000); respectively. Experiments were conducted at temperatures of 892, 1093, and 1292 K (measured by the vessel thermocouple) with reaction times of 15, 30, 60, 180, and 300 s.

At the start of each experiment, the furnace was heated until the vessel reached the desired temperature. Then, a 1 L tedlar sample bag (ESS GD0707-7000) was attached to the setup and the system was vacuumed with a vacuum pump (Edwards RV12) for 30 s with the sample valve and vacuum valve (both Swagelok SS-41GS2 ball valves) open while the feed valve and exhaust valve (both Parker 009-0089-900 solenoid valves) were closed to remove the majority of gases in the vessel and sample bag. A five-way valve (Swagelok SS-43ZF2-049) was then turned to select a nitrogen gas bottle (99.998% purity) regulated to 280 kPa and the vessel was purged with nitrogen by opening the feed valve and closing the sample valve and vacuum valve while the exhaust valve remained closed. The feed valve remained open for ∼ 1 s as the pressurized nitrogen purged the vessel and the tubing lines. The system was again vacuumed for 30 s by opening the vacuum and sample valve and closing the feed valve while the exhaust valve remained closed. This ensured that little to no residual gas would be left in the vessel and if there were residual gas it would be an inert gas.

To collect a sample, the sample valve was then closed but the vacuum valve remained open to continually vacuum the sample bag during the experiment. The five-way valve was turned to methane. The feed valve was opened, and methane (99.9% methane and 0.1% helium) pressurized at 400 kPa quickly filled the vessel. The time to fill the vessel (< 1 s) was always much less than the reaction time (≥ 15 s). After the vessel was filled, the feed valve was closed, and the gas was left in the vessel for the desired reaction time (between 15 s to 300 s).

The experiment was run with a microcontroller (Teensy) which controlled the timing of the two solenoid valves (feed and exhaust) while the other two ball valves were controlled manually. The valve voltages as well as the pressure and temperature of the system were measured using a computer program called LabVIEW from National Instruments Corporation. The data was collected using multiple data acquisition devices that were connected to a chassis (National Instruments cDAQ-9178). The solenoid valve voltages were collected using data acquisition device: NI-9223, the temperature data was collected using data acquisition device: NI-9213, and the pressure data was collected using data acquisition device: NI-9220. These data acquisition devices transferred the data into LabVIEW in intervals of 0.1 s.

After collecting the sample, the sample bag was removed from the experimental setup and attached to a gas chromatograph (GC) to be analyzed (not shown). The GC (Agilent 7890B) was first purged with nitrogen before measuring the sample gas. Nitrogen pressurized to 280 kPa was pushed through the GC for five minutes. Following the purge, a valve on the sample bag was opened, and a pump was turned on for 35 s forcing the sample gas to flow through the GC. The pump was then turned off and the ball valve before the pump was closed and remained closed until the pressure gauge read atmospheric pressure (to ensure the gas pressure in the GC column was at atmospheric pressure). The valve was then opened, and the GC was run to determine the concentration of methane, ethane, ethylene, propane, acetylene, benzene, carbon dioxide, oxygen, nitrogen, carbon monoxide, and hydrogen inside the sample bag. A calibration gas (49.56% methane and 50.45% hydrogen) was run through the GC daily before completing any experiments to ensure it was within calibration.

During each experiment carbon would accumulate on the walls of the reactor. The accumulation of carbon on the quartz vessel affected the reaction rate of the experiment depending on how much carbon accumulated on the walls of the vessel and the temperature of the experiment. Thus, after each experiment, the carbon accumulated on the vessel walls was removed by oxidizing the carbon with air. The vessel was cleaned by setting the furnace to 1073 K and injecting air pressurized to 262 kPa into the system. This was completed by turning the five-way valve to the air line and opening the feed valve for 1 s with all other valves closed. After a reaction time of 30 s the exhaust valve was opened for 1 s to release the products into the fume hood. This process was repeated 27 times before each experiment as lower temperatures or fewer cleaning cycles were found to be insufficient to remove all the accumulated carbon.

There is a minimum concentration of a species that a GC can reliably detect. The limit of detection, $L_{D}$, is the lowest measurable concentration, shown in Eq. (1), that can be distinguished from the noise when it is present in the sample and is quantified as [2],

(1) $$L_{D}=L_{B}+1.645\;\sigma_{L}$$

where,$\;L_{B}$ is the limit of blank and $\sigma_{L}$ is the standard deviation of the lowest calibrated concentration of a species [3]. $\sigma_{L}$ was calculated from the calibration data of the GC, specifically from calibration bottles with the lowest concentration of hydrocarbons that were used to calibrate the GC. The limit of blank, shown in Eq. (2), is the highest measured concentration of a species expected to be found when there is no species present in the sample [2]

(2) $$L_{B}=x_{blank}+1.645\;\sigma_{blank}$$

where, $x_{blank}$ is the mean value for all the blank concentrations, and $\sigma_{blank}$ is the standard deviation of the blank concentrations [3]. $x_{blank}$ and $\sigma_{blank}$ were determined by analyzing 4.8 purity nitrogen (99.998%) with the GC. These values were orders of magnitude smaller than the calculated $\sigma_{L}$ (>1000 times smaller) and therefore were negligible. Thus, the limit of blank is negligible in the calculation of the limit of detection. Table A1 shows the $L_{D}$, $\sigma_{L}$, number of calibrations used, and the calibration gas concentration for the species of interest.

The uncertainty in the measured concentration for each species, shown in Eq. (3), was determined using data from multiple experiments as well as the bias uncertainty from the calibration of the GC. The total uncertainty was determined from,

(3) $$U_{x}=\sqrt{P_{x}^{2}+B_{x}^{2}}$$

where, $U_{x}$ is the total uncertainty for each species, $P_{x}$ is the precision uncertainty and $B_{x}$ is the bias uncertainty from the calibration of the GC for each species.

The precision uncertainty, shown in Eq. (4), is defined as,

(4) $$P_{x}=t_{\frac{\propto }{2},\nu }\frac{\sigma_{x}}{\sqrt{N}}$$

where, N is the total number of experiments, $t_{\frac{\propto }{2},\nu }$ is the t-value for a confidence interval of 95%, ∝ is equal to one minus the confidence interval (1−C, where C=0.95), ν is the degrees of freedom (N−1), and $\sigma_{x}$ is the standard deviation in the measurement.

The bias uncertainty, shown in Eq. (5), is defined as,

(5) $$B_{x}=x_{\text{ave}}E$$

where, $x_{\text{ave}}$ is the average molar concentration of the species and E is the absolute value of the maximum relative difference, shown in Eq. (6), between the calibration gas concentration, $c_{a}$, and the value measured by the GC, $c_{m}$,

(6) $$E=\text{max}\left\{\left|\frac{c_{m_{i}}-c_{a_{i}}}{c_{a_{i}}}\right|:\;i=1..n\right\}$$

Table A2 shows the maximum relative differences for every species measured.

The total uncertainty in temperature and initial pressure were also calculated the same way as above. The difference being in the calculation of bias uncertainty. The bias uncertainty for temperature was 2.2 K or 0.75% of the temperature measurement, whichever was greater. The bias uncertainty for pressure was 0.5% of full scale (100 psi) which is 3.4 kPa. The uncertainty in time was estimated to be the time for the vacuum valve to be manually closed followed by the time for the sample valve to be manually opened after hearing the exhaust valve open. This time was estimated to be 3 s in a worst-case scenario.

A gas chromatograph (Agilent 7890B) was used to measure the molar concentration of the gas phase products. The GC has three channels, two thermal conductivity detectors (TCD) and one flame ionization detector (FID). The first TCD channel uses hydrogen as a carrier gas to measure the concentration of oxygen, nitrogen, carbon dioxide, and carbon monoxide. The second TCD channel uses argon as a carrier gas to measure the concentration of helium and hydrogen. The TCD channels measure the concentration of the sample by comparing the thermal conductivity of two flows, one with just the carrier gas and the other with the carrier gas and the sample. The difference in thermal conductivity between the flows determines which species and the molar concentration of the species with the carrier gas. Argon had to be used as a carrier gas instead of hydrogen in one of the TCD channels to measure the concentration of hydrogen. The detector temperature was 200 °C for both TCD channels and the carrier gas had a flow rate of 45 mL/min. The FID channel uses hydrogen as a carrier gas, nitrogen as a make-up gas, and air to ignite the hydrogen to measure the concentration of methane, ethane, ethylene, propane, acetylene, and benzene for this method. The FID channel measures the concentration of the sample by burning the sample mixed with the carrier gas with a hydrogen-air flame that burns the organic components to create ions. These ions are then collected and tested to determine the concentration of the hydrocarbons. The detector temperature was 523 K for the FID channel and hydrogen, air, and nitrogen had flow rates of 40 mL/min, 400 mL/min, and 25 mL/min, respectively. Table A3 shows the columns that are used in the GC to separate the components in the sample before sending them to the detectors to be analyzed.

Table A4 shows the retention times, area, response factor, standard number, and the mole fraction for each of the calibration gases used. Table A5 shows the relative differences for all measured species.

## Ethics Statement

Our work meets ethical requirements and does not involve human subjects, animal experiments, or any data collected from social media platforms.

## CRediT authorship contribution statement

**James Tatum:** Investigation, Data curation, Writing – original draft. **Ambuj Punia:** Methodology, Software, Formal analysis. **Larry Kostiuk:** Supervision. **Marc Secanell:** Resources, Supervision, Writing – review & editing. **Jason Olfert:** Project administration, Supervision, Writing – review & editing.

## Declaration of Competing Interest

The authors declare that they have no known competing financial interests or personal relationships that could have appeared to influence the work reported in this paper.

## Data Availability

Methane pyrolysis products in a constant-volume reactor as a function of time (Original data) (Mendeley Data).
